# Optical Coherence Tomography and Optical Coherence Tomography–Angiography Chronic Changes in End-Stage Renal Disease: A Systematic Review

**DOI:** 10.3390/diagnostics16030459

**Published:** 2026-02-02

**Authors:** Ioana-Madalina Bilha, Stefana Catalina Bilha, Nada Akad, Adrian Covic, Daniel-Constantin Branisteanu, Calina Anda Sandu, Vlad Constantin Donica, Camelia Margareta Bogdanici, Simona-Eliza Giusca, Irina Draga Caruntu

**Affiliations:** 1 Department of Morpho-Functional Sciences I-Histology and Pathology, “Grigore T. Popa” University of Medicine and Pharmacy, 700115 Iasi, Romania; madalinabilha@gmail.com (I.-M.B.);; 2 Department of Internal Medicine II-Endocrinology, “Grigore T. Popa” University of Medicine and Pharmacy, 700115 Iasi, Romania; 3 Department of Internal Medicine II-Nephrology, “Grigore T. Popa” University of Medicine and Pharmacy, 700115 Iasi, Romania; 4 Department of Surgery II-Ophthalmology, “Grigore T. Popa” University of Medicine and Pharmacy, 700115 Iasi, Romaniavlad-constantin.donica@umfiasi.ro (V.C.D.); camelia.bogdanici@umfiasi.ro (C.M.B.); 5 Romanian Medical Science Academy, 030171 București, Romania

**Keywords:** optical coherence tomography, end-stage renal disease, retinal microvasculature

## Abstract

**Background/Objectives**: End-stage renal disease (ESRD) is characterized by profound and progressive microvascular dysfunction that contributes significantly to systemic morbidity. Because the retinal and renal microcirculations share structural and physiological similarities, optical coherence tomography (OCT) and OCT angiography (OCTA) have emerged as promising tools for detecting ocular microvascular changes that may parallel systemic vascular injury. This systematic review aimed to consolidate evidence on chronic retinal and choroidal alterations in ESRD as assessed by OCT and OCTA. **Methods**: A systematic search of PubMed/MEDLINE (inception–June 2025) was performed using combinations of terms related to OCT, OCTA, ESRD, and hemodialysis. After removing duplicates and screening titles, abstracts, and full texts, we included clinical studies involving adults with ESRD or undergoing dialysis that reported chronic or baseline OCT/OCTA findings. Non-English publications, editorials, conference abstracts, case reports, and studies limited to acute pre-/post-dialysis changes were excluded. Seventeen studies met eligibility criteria. Acute findings were summarized narratively only when no chronic data existed for a specific parameter but were not incorporated into the primary synthesis. **Results**: Across eligible studies, chronic structural and perfusion abnormalities were consistently reported, including thinning of the retinal nerve fiber and ganglion cell layers, reduced macular and peripapillary vascular densities, enlarged foveal avascular zones, and decreased choroidal thickness. These alterations aligned with markers of disease severity and systemic microvascular burden. **Conclusions**: Retinal imaging reveals reproducible chronic microvascular changes in ESRD and may serve as an accessible adjunct for systemic vascular assessment. We highlight the potential significance of retinal vascular screening in this population and the need for more standardized imaging protocols to support the effective integration of retinal biomarkers into CKD diagnostic and monitoring strategies.

## 1. Introduction

**Figure 1:**
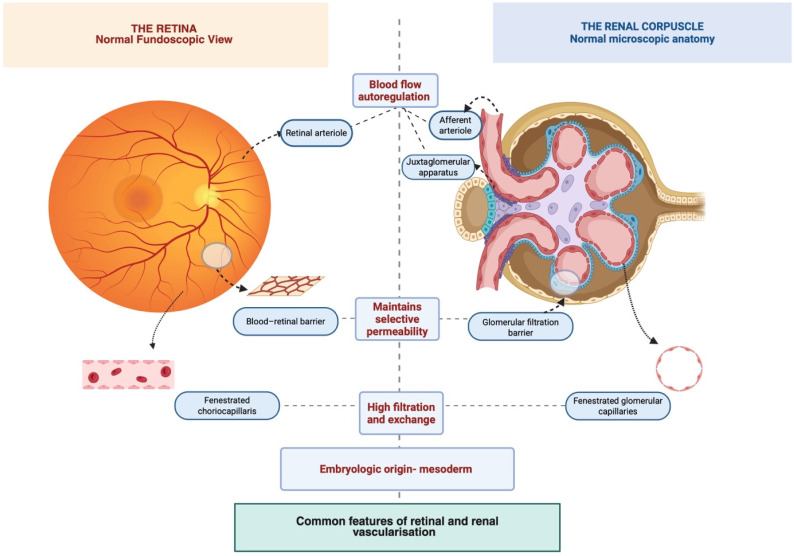
Common features of retinal and renal vascularization.

Chronic kidney disease (CKD) is a progressive systemic disorder marked by microvascular and neurodegenerative injury that extends beyond the kidney [1,2]. The retina and choroid are both highly vascularized tissues particularly sensitive to endothelial dysfunction and hemodynamic instability. Because the retina and choroid share microvascular features with the renal circulation (Figure 1), they provide a practical, noninvasive window into systemic microangiopathy [3].

Advances in optical coherence tomography (OCT) and OCT angiography (OCTA) allow high-resolution quantification of the neuroretinal structure (e.g., retinal nerve fiber layer (RNFL), ganglion cell layer (GCL), inner plexiform layer (IPL), macular thickness) and layer-specific perfusion metrics (e.g., superficial vascular plexus (SVP)/deep vascular plexus (DVP) vessel density, foveal avascular zone (FAZ), choriocapillaris flow deficits). These biomarkers are promising for early detection, monitoring, and risk stratification in CKD; however, published findings vary with CKD stage, dialysis status, scan timing, and device/segmentation protocols [2,3,4].

In end-stage renal disease (ESRD), hemodialysis (HD) introduces distinctive physiologic stressors—ultrafiltration, osmotic shifts, and blood pressure variability—that can acutely modulate ocular perfusion and thickness measures, particularly within the choroid. Two recent meta-analyses [5,6] converge on modest but consistent post-HD subfoveal choroidal thinning, with minimal acute change in retinal thickness parameters and larger effects in eyes with proliferative diabetic retinopathy—highlighting the choroid’s heightened susceptibility to systemic fluid shifts [5,6].

Longer-term (chronic) ocular manifestations of CKD include retinal arteriolar narrowing, choroidal thinning, and RNFL/GCIPL loss [7,8,9,10], driven and often amplified by comorbid diabetes mellitus (DM) and hypertension [8,9,10]. These structural alterations correlate with declining eGFR and albuminuria, and thus better capture cumulative burden of uremia, hypertension, and inflammation. Because these effects are often amplified by DM, it is essential to stratify findings by DM status and to distinguish disease-related trajectories from transient peri-dialytic fluctuations [10].

This systematic review aims to summarize OCT and OCT-A evidence in patients with ESRD (stage 5 CKD or undergoing HD), explicitly stratified by the presence or absence of DM.

## 2. Materials and Methods

**Figure 2:**
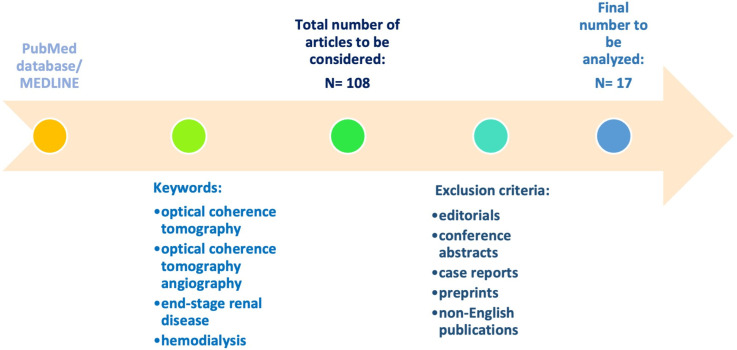
Study design.

We systematically searched PubMed/MEDLINE from database inception to June 2025 using combinations of the following terms and their synonyms/MeSH where applicable: *optical coherence tomography*, *optical coherence tomography angiography*, *end-stage renal disease*, and *hemodialysis*, combined with Boolean operators. The search yielded 108 records. After duplicate removal and screening of titles/abstracts followed by full texts, we excluded editorials, conference abstracts, case reports, preprints, and non-English publications. We limited inclusion to clinical studies in adults (≥19 years) with ESRD and/or undergoing dialysis that reported chronic/baseline (i.e., not immediate pre-versus post-dialysis) changes in OCT and/or OCTA parameters. Applying these criteria, 17 studies were deemed eligible for the review. A schematic illustration of the study design is presented in Figure 2.

In the existing literature, several OCT parameters are reported exclusively as acute, session-related (pre/post hemodialysis) changes with no corresponding evidence of chronic alterations (e.g., lamina cribrosa). For outcomes lacking chronic data, we extracted the available acute findings and summarized them narratively to provide context, while excluding them from the primary synthesis focused on chronic effects.

## 3. Results

### 3.1. OCT Parameters—Structural Changes in Non-Diabetic ESRD Patients

#### 3.1.1. Retinal Structure and Choroid Thickness

Most studies evaluating OCT findings in CKD focus on the macular and peripapillary regions. Table 1 summarizes key OCT-based studies regarding structural retinal and choroidal changes in non-diabetic ESRD patients reported in nine observational studies (six cross-sectional and three prospective); where studies included mixed diabetic and non-diabetic cohorts [11,12,13,14], we reported key findings pertaining to non-diabetic patients. When subgroup-specific data were unavailable, findings were recorded as mixed-cohort (pooled) results [12,14]. Sample sizes ranged from 20 to 171 participants, spanning non-diabetic ESRD/HD cohorts and mixed CKD 2–5 populations. All studies [11,12,13,14,15,16,17,18] employed structural OCT, while one study [19] additionally incorporated OCTA and color fundus photography; however, given the structural focus of this section, we included only the correlations between fundus imaging findings and retinal and choroidal thickness parameters obtained by structural OCT. The focus point of most studies using OCT for retinal screening in CKD patients is the RNFL due to its high specificity for retinal neurodegeneration as an end-organ lesion. A reduction in RNFL thickness may reflect neural loss in the central nervous system and its quantification is relevant when neurodegeneration is suspected. Apart from the RNFL, most OCT assessments included in studies also encompass a composite layer consisting of the GCL and IPL, serving as a representation of the inner retina.

Significant reductions in RNFL thickness in dialysis patients compared to healthy controls have been reported since 2009, irrespective of dialysis type [17]. Because the study of Demir et al. [17] excluded patients with DM, the observed RNFL thinning likely reflects non-diabetic mechanisms (e.g., uremic toxins, chronic ischemia, anemia, hypertension) rather than diabetic optic neuropathy. These findings highlight the importance of conducting comprehensive ocular assessments in patients with ESRD to avoid potential misdiagnosis of glaucoma [17].

A study from Tang et al. [19] demonstrated a direct association between the extent of fundus damage, retinal thickness and CKD severity, underscoring the clinical relevance of regular fundus examinations in this patient population [19]. A higher risk for fundus damage was found in men (OR = 8.52, *p* < 0.001 compared to females) and with increasing cholesterol levels (OR = 1.995, *p* = 0.001 for each unit increase), while a higher hemoglobin concentration appeared to be protective (OR = 0.964, *p* = 0.033 for each unit increase) [19].

Yeung et al. [15], in a two-year longitudinal case–control study involving 152 CKD 3-5D eyes, observed a linear pattern of pRNFL reduction that was independently associated with CKD severity, rim area, and hypertension [15].

More detailed segmentation methods that include volumetric analysis may offer greater sensitivity for detecting neuroretinal damage. Wu et al. [12] have included 171 CKD patients in a case–control study. They reported significant thinning of both peripapillary RNFL (pRNFL) and macular ganglion cell complex (GCC), together with an increase in global loss volume (GVL) and focal loss volume (FVL), respectively, with worsening CKD. The study highlights a potential link between declining kidney function and retinal neurodegeneration, although causality cannot be inferred from the study design [12].

In a single-center cross-sectional study of 32 ESRD patients undergoing maintenance HD, Jung et al. [13] measured both RNFL thickness and retinal layer volumes. They found a reduction in the GCL and GCL-inner plexiform layer (GCL-IPL) volume and of the RNFL thickness in the temporal superior sector after excluding diabetic patients from the analysis, suggesting that neurodegenerative retinal changes in ESRD are not solely attributable to diabetic microvascular disease. The study did not identify significant associations between duration of dialysis and retinal layer thickness or volume, implying that neuroretinal alterations may be more closely related to the systemic effects of ESRD itself rather than cumulative time on dialysis [13]. Nevertheless, Hong et al. [16] demonstrated that RNFL thickness reduction is significantly related to HD vintage, and the relationship appears to be independent of intraocular pressure (IOP) elevation [16].

On the contrary, Atilgan et al. [18] failed to show a significant association between HD vintage and peripapillary RNFL thickness in a non-diabetic ESRD cohort. However, the RNFL thinning, reflecting neurodegenerative loss, was consistent within 6 months of follow-up, despite transient post-HD increase. The initially reported decrease in macular thickness pre-HD was not sustained at 6 months, implying short-term fluid/pressure effects and mandating cautious glaucoma/fluid-status interpretation in these patients. Macular volume also failed to show an association with reduced eGFR, but rather reflected the presence of DM and ischemic heart disease in the study of Mustafar et al. [14]

The choroid is a vascular layer situated between the retina and the sclera, playing an essential role in supplying oxygen and nutrients to the outer retina, particularly the retinal pigment epithelium (RPE) and photoreceptors. Studies have failed to show chronic choroidal thickness changes across CKD stages [19]. However, Chen et al. [11] reported patients with DM tend to show reduced peripapillary RNFL thickness and greater choroidal thinning compared to non-diabetic patients, likely reflecting milder retinal structural changes in the absence of DM [11].

#### 3.1.2. Lamina Cribrosa (LC)

The LC is a specialized sieve-like structure within the optic nerve head that serves as a critical biomechanical and physiological interface between the intraocular and intracranial compartments. It is composed of collagenous connective tissue beams lined by astrocytes, through which retinal ganglion cell axons exit the eye to form the optic nerve [20]. This structure provides both mechanical support and metabolic exchange for the traversing axons while maintaining the pressure gradient between the intraocular and retrobulbar spaces. Alterations in the LC’s architecture—whether due to elevated intraocular pressure, systemic vascular dysregulation, or disrupted translaminar pressure gradients—can lead to axonal deformation, impaired axoplasmic flow, and compromised perfusion of the optic nerve head. Such biomechanical stress is considered a central pathogenic mechanism in glaucomatous optic neuropathy, linking structural deformation of the LC to functional visual field loss [21].

Despite the absence of literature data reporting chronic OCT changes in LC in ESRD patients, the prospective observational study performed by Kim et al. [22], investigating the acute effects of HD on LC in 29 HD patients, is of interest [22]. Their findings revealed a post-dialysis reduction in mean ocular perfusion pressure and a decrease in the anterior depth of the LC, despite the absence of significant IOP changes. Because anterior displacement of the LC is a characteristic feature of glaucomatous eyes, this study provides novel evidence suggesting a potential mechanism underlying the increased risk of glaucoma observed in patients undergoing HD.

These findings suggest that rapid fluid and pressure shifts during dialysis temporarily alter optic nerve head biomechanics, potentially contributing to chronic optic nerve vulnerability in patients with end-stage kidney disease. However, testing this hypothesis requires longitudinal studies.

### 3.2. OCTA Changes in Non-Diabetic HD Patients: Retinal and Choriocapillaris Vascular Parameters

The retinal and choroidal vasculature provide a unique window into systemic microvascular health. OCTA reports layer-specific metrics that capture microvascular integrity and perfusion: vessel/perfusion density in the SVP and DVP, respectively, and FAZ area/circularity. In ESRD and during HD, these measures help distinguish chronic neurovascular injury (e.g., reduced macular densities, enlarged FAZ) from acute HD-related hemodynamic/osmotic effects, which often manifest most clearly at the choriocapillaris [23,24,25]. Table 2 summarizes studies describing chronic retinal and choroidal perfusion alterations in non-diabetic patients with ESRD. This subgroup includes four cross-sectional evaluating retinal microvasculature in ESRD using OCTA and/or fundus imaging. Sample sizes ranged from 84 to 200 patients, focusing on non-diabetic ESRD, but three [12,14,15] of them also included diabetic ESRD patients. In studies with mixed diabetic and non-diabetic cohorts, findings were recorded as mixed-cohort (pooled) results when subgroup-specific data were unavailable [12,14].

A study from Mustafar et al. [14], found a direct correlation between increased retinal vascular tortuosity and lower eGFR levels [14]. In a large cross-sectional cohort of 200 CKD stage 3–5 patients (including 76 ESRD—27 on HD, 33 on PD) assessed with OCTA, Yeung et al. [26] also reported significant retinal microvascular rarefaction: vessel density was reduced in both the superficial and deep vascular plexuses across all quadrants (*p* < 0.001), and the FAZ showed altered morphology with a higher circularity index (*p* = 0.001) versus controls. These changes become more pronounced with advancing CKD and aging in both diabetic and non-diabetic patients. However, CKD patients with DM had a trend towards greater SVP and DVP reduction compared to non-diabetic CKD patients [26].

Moreover, retinal and choroidal plexuses exhibit differential chronic responses to ESRD. In non-diabetic CKD stages 2–5, OCTA retinal vessel density increased with progression toward ESRD, whereas the choroidal vascular index was unchanged across stages. This pattern suggests compensatory retinal vasodilation or measurement artifact, with comparatively stable choroidal vasculature across CKD severity [19].

Complementing these observations, Wu et al. [12] showed that lower SVP vessel density independently associates with macular thinning—even after adjusting for age, sex, diabetes, and hypertension—supporting impaired neurovascular coupling in CKD [12].

### 3.3. Microvascular Alterations in Diabetic CKD Patients: Insight from OCT and OCTA Imaging

One of the most common comorbidities among patients with CKD is DM, with diabetic nephropathy being among the main factors in developing ESRD [27]. It is hard to establish a study with a lot of patients with diabetes and CKD and clearly state what retinal microvascular changes are attributed to each disease. Despite microvascular changes being difficult to quantify within the kidney, retinal small vessel imaging may offer a great perspective on systemic microvascular modifications. Table 3 summarizes the data from the literature describing chronic retinal and choroidal microvascular changes in diabetic patients with ESRD. This group includes four observational studies (two prospective [28,29], two retrospective [30,31]) in ESRD patients with diabetic retinopathy evaluating the impact of HD initiation or long-term HD on macular and choroidal structure using structural OCT and OCTA. Sample sizes ranged from 15 to 70 patients (26–132 eyes) (Table 3).

In ESRD patients with diabetic retinopathy on long-term HD, OCTA shows widespread ischemia (universal nonperfusion, enlarged FAZ) and persistent pathology (cystoid diabetic macular edema (diabetic macular edema (DME), epiretinal membranes(ERM)) despite stable macular thickness, as reported by He et al. [28].

Macular edema in ESRD is multifactorial and highly dependent on systemic factors such as blood urea nitrogen (BUN), eGFR, hypertension, anemia, diabetes severity, and glycemic control (HbA1c). Hwang et al. [29], in a study including 26 eyes from 15 ESRD patients with DM, found that initiating dialysis in diabetic ESRD is associated with a significant reduction in macular edema and central subfield thickness, likely reflecting relief of uremia/volume overload rather than ocular treatment effects. Takamura et al. [30] also showed that initiating HD in ESRD patients with diabetic retinopathy/DME yields sustained anatomical and functional benefits, and their OCT-based subtyping adds practical prognostic value: eyes with DME accompanied by subretinal detachment (SRD) experienced greater central retinal thickness reduction than those without SRD, indicating that OCT phenotype can help predict the degree of edema resolution after starting dialysis. SRD, reported in 20.3% of eyes in their series, displayed a particularly significant response to HD initiation: OCT scan documented progressive resolution of subretinal fluid—seen in 25.0%, 41.7%, and 58.3% of SRD eyes at 1, 3, and 6 months, respectively—with complete disappearance in all eyes by 9–12 months, despite most receiving no ocular DME therapy. While the precise mechanism remains uncertain, the authors hypothesize that HD may restore fluid dynamics across the retina–choroid interface, enhancing mutual fluid exchange and thereby facilitating absorption of excess subretinal fluid; importantly, these structural gains paralleled improvements in vision and were independent of baseline laboratory values [30].

These findings highlight DM as a key modifier of ocular microvascular integrity in renal disease and underscore the value of OCT and OCTA for early detection of systemic microangiopathy.

Nonetheless, a prospective multicenter cross-sectional OCT study of ESRD patients compared diabetic vs. nondiabetic eyes over 2 weeks after starting HD. The DM group showed significantly larger choroidal reductions in subfoveal choroidal thickness (−13.3% vs. −9.5%, *p* = 0.049), large choroidal vessel layer thickness (−14.5% vs. −9.2%, *p* = 0.02) and subfoveal choroidal area (−21.9% vs. −17.2%, *p* = 0.032) compared to the non-diabetic group. Overall, HD induced more pronounced structural shrinkage—especially in the luminal (vascular) component—in diabetic eyes, suggesting heightened choroidal vascular dysfunction in DM [31].

## 4. Discussion

The increased prevalence of vascular disease among patients with CKD underscores the necessity for advanced vascular screening strategies, aimed at reducing the burden of ESRD management and improving overall survival outcomes [32]. Given the strong similarity between ocular and renal vascularization, assessing retinal microcirculation provides valuable insight into systemic microvascular alterations throughout the body [33].

The widespread availability of OCT and OCTA in ophthalmology departments worldwide makes these techniques valuable tools for retinal vascular imaging. In recent years, many studies have examined retinal structural changes in patients with systemic diseases, aiming to identify relevant biomarkers using this noninvasive imaging method. Understanding how retinal structure evolves in relation to CKD progression is particularly important for prevention and for defining personalized intervals for ophthalmologic follow-up [34].

In non-diabetic and/or mixed CKD and ESRD cohorts, most studies converge on a pattern of inner retinal thinning—particularly at the level of the RNFL and GCL—that parallels kidney dysfunction and, in some reports, dialysis duration [11,12,13,14,15,16,17,18,19,26]. Early work identified significant RNFL thinning in HD patients even in the absence of diabetes, suggesting that factors such as uremia, chronic ischemia, anemia, and hypertension may underlie neuroretinal loss rather than diabetic optic neuropathy alone [17]. Subsequent studies have linked advanced CKD with greater fundus damage and reduced retinal thickness, with male sex, dyslipidemia, and lower hemoglobin emerging as additional risk factors [19]. Both longitudinal and cross-sectional OCT analyses consistently show a CKD severity–dependent decline in pRNFL, accompanied by thinning of the macular GCC and increases in global and focal loss volumes across CKD stages [12,19,26]. Detailed layer-by-layer assessments in non-diabetic ESRD cohorts further demonstrate GCL and GCL–IPL volume loss, supporting a primary role of ESRD-related systemic factors in driving neurodegeneration [13]. While some studies have not identified a clear association between dialysis vintage and retinal layer metrics [13], others report a relationship between longer HD duration and RNFL thinning, partly mediated by intraocular pressure, and confirm progressive RNFL loss over follow-up even when a direct vintage effect is not evident [16].

A reduction in pRNFL and GCL thickness, as reported by Wu et al. [12], together with macular thinning and lower SVP density, supports the retinal neurovascular unit (NVU) concept, which encompasses the coordinated interaction of neurons, glia, and vessels to maintain retinal homeostasis [12]. Layer-specific volumetric analysis refines this assessment; for example, inner plexiform layer volume reduction was positively correlated with lower RNFL thickness in CKD patients, as shown by Jung et al. [13]. Microvascular disruption within the NVU leads to retinal thinning and functional decline and may reflect systemic vascular pathology [35].

Retinal NVU dysfunction is increasingly investigated as a potential early biomarker in neurodegenerative diseases [36]. CKD is associated with an increased risk of cognitive impairment [37]. In this context, although Peng et al. [38] did not find a significant association between RNFL thickness, GCC, and cognitive performance in 177 patients with CKD stage ≥3, they reported a positive correlation between reduced DVP density and subclinical cognitive decline, suggesting a potential role for DVP alterations as early screening biomarkers [38]. Nevertheless, further studies are needed to confirm these findings and to better define the clinical utility of OCT/OCTA-derived parameters in this setting.

By contrast, macular and choroidal metrics appear more variable and more strongly modulated by fluid status, angiogenic imbalance, and cardiometabolic comorbidities than by CKD stage alone. Atilgan et al. [18] noted that macular thinning observed pre-HD was not sustained at six months, suggesting that part of the macular signal reflects short-term shifts in volume and pressure rather than fixed structural damage. Similarly, Mustafar et al. [14] found that macular volume reductions were more closely linked to diabetes and ischemic heart disease than to eGFR, indicating that macular changes may preferentially capture superimposed cardiometabolic pathology. Large-scale data have not consistently demonstrated chronic choroidal thinning across CKD stages [19]; nonetheless, Nakano et al. [31] report significant choroidal structure changes after HD initiation, with greater reductions in subfoveal choroidal thickness and large-vessel layer thickness in diabetic than in non-diabetic patients. Thus, diabetic HD patients seem to have a more fragile or compromised choroidal circulation, so their outer retina (which depends on the choroid for blood supply) may be more vulnerable to underperfusion and damage than in non-diabetic HD patients [31]. These alterations, together with chronic retinal hypoperfusion, may manifest clinically as progressive visual decline, higher risk of diabetic retinopathy, and even acute vision loss in ESRD patients on HD [39].

DM is one of the leading causes of CKD and plays a critical role in the progression of systemic microvascular dysfunction. The coexistence of DM and CKD exacerbates endothelial injury, capillary dropout, and impaired autoregulation within both renal and ocular microcirculations [40]. Persistent hyperglycemia leads to oxidative stress, inflammation, and basement membrane thickening, resulting in progressive capillary loss and vascular leakage [41]. In CKD patients, these processes are further amplified by uremic toxins, anemia, and systemic hypertension, which exacerbate endothelial dysfunction and impair tissue perfusion [42]. OCT and OCTA studies have demonstrated significant reductions in retinal vascular density, enlargement of the FAZ, and thinning of the retinal nerve fiber and choroidal layers in diabetic CKD populations [26]. Liu et al. [43]. examined 1408 diabetic patients and identified a positive correlation between impaired renal function, reduced pRNFL thickness, and altered retinal microcirculation [43].

Severe microvascular damage is present in diabetic ESRD patients on long-term HD despite apparently “stable” macular thickness: OCTA data show advanced diabetic retinopathy with widespread non-perfusion, abnormal microvasculature, enlarged FAZ, and frequent cystoid DME and ERM, indicating that OCT-based thickness alone underestimates disease burden and that HD does not reverse underlying ischemia or neurodegeneration, which are largely driven by long-standing diabetes and structural microvascular remodeling [28].

Longitudinal studies indicate that initiation of HD in diabetic patients improves uremia and volume overload, resulting within weeks to months in reduced central retinal and choroidal thickness, decreased macular edema, and improved visual acuity, particularly in SRD-type DME. Although HD effectively alleviates fluid-related retinal and choroidal congestion, it does not reverse chronic microvascular ischemia; systemic uremia and fluid status remain key determinants of DME, and choroidal parameters may serve as sensitive markers of circulatory and volume changes. Clinically, HD provides meaningful anatomical and functional benefits in ESRD patients with DR/DME, but continued retinal surveillance is required, as persistent ischemia and advanced disease often necessitate intravitreal therapy.

While effective in removing metabolic waste and excess fluid, HD induces rapid hemodynamic and osmotic fluctuations that can affect systemic and ocular microcirculation [33]. Acute retinal and choroidal structural changes following HD have been reported. Chen et al. [11] observed increased macular RNFL thickness and reduced choroidal thickness one hour after HD compared with pre-dialysis measurements, likely reflecting rapid volume and osmotic shifts [11]. Multiple OCT/OCTA studies have similarly demonstrated post-dialysis reductions in subfoveal choroidal thickness, particularly in the temporal and nasal regions, and a pooled self-controlled case series by Su et al. [6] confirmed a consistent moderate decrease across studies [6]. Additional reports described increased choriocapillaris non-perfusion after HD, with largely unchanged retinal vascular densities, suggesting greater choroidal sensitivity to HD-induced stress [11,44,45]. These changes are more pronounced in diabetic ESRD patients, likely due to pre-existing diabetic choroidopathy and impaired vascular autoregulation, which amplify the impact of HD-related circulatory stress [6,30,31,46,47].

Posterior bowing of the optic nerve—posterior displacement of the LC driven by altered translaminar pressure gradients or elevated IOP—can compromise axonal support and perfusion, contributing to glaucomatous damage [48]. In CKD, volume changes during HD may promote such alterations and, together with RNFL thinning, make it more difficult to distinguish dialysis-related changes from true glaucomatous damage. Comprehensive evaluation, including diurnal IOP profiling and pre- and post-dialysis measurements, is therefore crucial for accurate diagnosis and management in this population.

OCTA shows great promise when integrated into artificial intelligence (AI) models, building on existing work where deep learning on color fundus photography has predicted CKD/diabetic kidney disease risk and incident kidney failure (including retinal age gap and Reti-CKD scores) and OCT-based models have already classified diabetic nephropathy with high accuracy, sensitivity, and specificity [49].

Overall, the available evidence suggests that retinal structure in CKD—especially in patients on HD—is influenced by both chronic neurodegenerative changes related to kidney dysfunction and its comorbidities and superimposed acute, dialysis-related fluid and perfusion shifts. Further longitudinal studies are needed to determine whether repeated HD-induced choroidal alterations contribute to long-term structural or functional ocular impairment. Interpretation of OCT metrics in this population should account for underlying disease severity and vascular comorbidities, as well as the timing of imaging in relation to dialysis sessions, given that transient peri-dialysis retinal and choroidal changes may mask or mimic true progressive neurovascular injury, especially in patients with DM. Given the high prevalence of hypertension in ESRD, hypertension-associated vascular remodeling and impaired autoregulation may further modify retinal and choroidal microcirculation, and thus should be accounted for when interpreting imaging findings.

Finally, it should be noted that although recruitment strategies were generally reported, detailed retinopathy grading or comprehensive clinical retinal findings were available in only a few cases, which may limit interpretation of baseline retinal status. In addition, the severe systemic disease present in all included patients and the high prevalence of arterial hypertension act as significant confounding factors, complicating the establishment of a direct and independent association between ocular findings and renal dysfunction per se. Furthermore, variability in inclusion and exclusion criteria regarding systemic comorbidities may contribute to heterogeneity across studies.

## 5. Conclusions

In summary, the available OCT reports most consistently indicate chronic retinal and choroidal changes in ESRD—particularly inner retinal thinning at the level of the RNFL and GCL/GCC and NVU disruption—with greater changes reported in advanced disease/longer HD duration, and in the presence of DM. These findings are compatible with cumulative systemic microvascular/neurovascular injury, although confounding by comorbidities (such as arterial hypertension) and dialysis-related hemodynamic shifts cannot be excluded. In contrast, macular and choroidal metrics are more variable and may be influenced by fluid status and angiogenic imbalance.

Overall, OCT (and OCTA, where available) appears to be useful for characterizing ocular involvement in CKD/ESRD and may hold promise as adjunct imaging biomarkers of broader neurovascular burden. Well-designed longitudinal studies are needed to define prognostic value for assessing renal and vascular progression and cognitive decline.

## Figures and Tables

**Table 1:** OCT/OCTA studies reporting chronic structural changes in cohorts focusing on non-diabetic ESRD patients.

Study (Year)	Study Design/Population (n)	Mixed Cohort (Included Patients with DM)	Other Systemic Comorbidities	Imaging Modality	Main Retinal Parameter(s)	Key Findings	Clinical Implication
Demir et al. (2009) [17]	Cross-sectional case–control; n = 33 ESRD (n = 18 HD and n = 15 PD), (66 eyes)	No	Hypertension (n = 15)	OCT	RNFL thickness	Global and quadrant RNFL significantly thinner in ESRD compared to healthy controls (*p* < 0.05). No differences in RNFL between HD and PD or in predialysis compared to postdialysis.	Evidence of RNFL thinning in ESRD in the absence of DM.
Atilgan et al. (2016) [18]	Prospective observational study; n = 20 HD patients (40 eyes)	No	Not reported	FD-OCT	RNFL thickness Macular thickness	Baseline (pre-HD): Temporal, inferior, and average RNFL thinner vs. controls (*p* < 0.015); macular thickness thinner vs. controls (*p* < 0.001). Only macular thinning correlated with HD duration. Post-HD changes: Transient increases in RNFL and macular thickness at day 1 and month 1; effects not sustained at 6 months except a consistent increase in superior quadrant RNFL.	Evidence of RNFL thinning in ESRD in the absence of DM.
Chen et al. (2018) [11]	Cross-sectional, n = 90 eyes from n = 45 HD patients	Yes n = 6 DM	Hypertension (n = 9)	OCT	Choroidal thickness RNFL	No significant difference compared to controls in the overall mixed cohort. Decreased choroidal thickness and RNFL in DM compared to non-diabetic HD patients.	Patients with non-diabetic causes of kidney failure often exhibit milder retinal structural changes.
Jung et al. (2020) [13]	Cross-sectional; n = 32 HD patients	Yes n = 7	Not reported	SD-OCT layer segmentation	GCL volume, IPL volume, IRL volume and RNFL thickness	Significant lower GCL volume (*p* = 0.014), GCL-IPL volume (*p* = 0.024) and temporal superior RNFL (*p* = 0.021) in non-diabetic HD.	Severe neurodegenerative retinal alterations in non-diabetic HD patients compared to healthy controls (neurodegenerative retinal changes independent of diabetic microvascular disease).
Wu et al. (2020) [12]	Case–control, n = 171 CKD 2–5 (n = 21 HD, n = 29 PD)	Yes n = 73	Hypertension (n = 154), Cardiovascular disease (n = 38), Gout (n = 10)	SD-OCT OCTA	GCC GLV FLV PfRT RNFL	Significantly decreased GCC, RNFL and increased GLV and FLV with CKD progression (*p* < 0.005 for all), with greatest loss in advanced disease in the overall mixed cohort. PfRT decrease strongly corelated with retinal neural impairment in the mixed overall cohort (*p* < 0.001).	Significant reduction in macular thickness and retinal neural parameters in mixed ESRD cohort (diabetic and non-diabetic).
Yeung et al. (2022) [15]	Prospective cohort study, n = 152 eyes CKD stages 3–5 D (n = 42 ESRD undergoing dialysis)	No	Hypertension (n = 66)	SD-OCT	pRNFL	No significant difference in pRNFL in the overall CKD eyes compared to controls. Accelerated annual RNFL thinning in CKD stage 4–5 and ESRD compared with healthy controls (*p* = 0.009 for both, respectively); correlated with eGFR decline. CKD stage was negatively associated with 2-year change in pRNFL thickness (*p* = 0.01).	Advanced CKD is associated with faster pRNFL decline compared to healthy aging. Hypertension, severity of CKD, and rim area are independent predictors of pRNFL loss over 2 years in patients with CKD.
Mustafar et al. (2023) [14]	Cross-sectional; n = 84 patients stages 3–5 CKD (n = 28 ESRD not undergoing dialysis)	Yes N = 54	Hypertension (n = 78), Ischemic heart disease (n = 20)	Fundus imaging + OCT	Macular volume	Macular volume did not correlate with CKD progression, but negatively correlated with DM (r = 0.0015, *p* = 0.04) and lower in patients with ischemic heart disease (*p* = 0.038) in the overall cohort.	Reduced macular volume rather reflects DM and CVD risk than CKD progression in mixed CKD diabetic and non-diabetic cohort.
Hong et al. (2024) [16]	Cross-sectional; n = 60 HD patients (n = 119 eyes)	No	Cardiovascular disease (n = 10)	SD-OCT	RNFL thickness	Longer dialysis duration associated with greater RNFL thinning in the temporal and inferior quadrants (*p* < 0.01).	Duration-dependent retinal neurodegeneration.
Tang et al. (2024) [19]	Cross-sectional, study lot of n = 118 CKD 2-5 (n = 48 ESRD), OCTA exam for n = 72 patients (n = 142 eyes), eyes from ESRD patients: n = 68	No	Not reported	Fundus imaging + OCT/OCTA	Fundus retinopathy grade Retinal thickness Choroidal thickness	Severity of fundus damage and retinal thinning correlated with CKD stage (Kendall’s tau-b = 0.494, *p* < 0.001). Risk of fundus injury reduced by 94.8% in CKD stage 3 compared to stage 5. Choroidal thickness did not differ significantly across CKD stages.	Fundus pathology reflects renal decline.

Abbreviations: *CKD* chronic kidney disease; *CVD* cardiovascular disease; *DM* diabetes mellitus; *eGFR* estimated glomerular filtration rate; *ESRD* end-stage renal disease; *FD-OCT* Fourier domain optical coherence tomography; *FLV* focal loss volume; *GCC* macular ganglion cell complex; *GLV* global loss volume; *HD* Hemodialysis; *OCT* Optical Coherence Tomography; *OCTA* Optical Coherence Tomography Angiography; *PD* perfusion density; *PfRT* parafoveal retinal thickness; *pRNFL* Peripapillary Retinal Nerve Fiber Layer; *RNFL* Retinal Nerve Fiber Layer.

**Table 2:** Quantitative Assessment of Chronic Retinal and Choroidal Perfusion in non-diabetic ESRD patients.

Study (Year)	Study Design/Population (n)	DM	Other Systemic Comorbidities	Imaging Modality	Main Retinal Parameter(s)	Key Findings	Clinical Implication
Yeung et al. (2019) [15]	Cross-sectional; 200 CKD stages 3–5 patients (n = 76 ESRD, among which n = 27 HD and n = 33 PD), control group (n = 50 eyes).	N = 91 in the overall group	Hypertension (n = 66)	OCTA	SVP DVP FAZ size, perimeter and circularity index	In the overall cohort, CKD patients exhibited reduced vessel density in both plexuses (*p* < 0.001 for all quadrants) and enlarged FAZ circularity index (*p* = 0.001) compared to controls; changes correlated with declining eGFR and increased serum creatinine. Age, DM and CKD stage are negatively associated with SVP (*p* < 0.001 for all) and DVP (*p* = 0.002 for age, *p* = 0.005 for DM and *p* = 0.003 for CKD stage), respectively (overall cohort). CKD patients with DM had a trend towards greater SVP and DVP reduction compared to non-diabetic CKD patients.	Significant rarefaction of retinal microvasculature in both SVP and DVP with advancing CKD, irrespective of DM.
Wu et al. (2020) [12]	Case–control, n = 171 CKD 2-5 (n = 21 HD, n = 29 PD)	N = 62	Hypertension (n = 154), Cardiovascular disease (n = 38), Gout (n = 10)	SD-OCT OCTA	GCC GLV FLV PfRT RNFL SVP-VD DVP-VD	Structural neural parameters (PfRT, GCCt, GLV, FLV, RNFLt) were significantly correlated with SVP-VD (*p* < 0.001) but not with DVP-VD (all *p* > 0.1) in the overall cohort.	Retinal neural parameters were associated with the severity of CKD and correlated with the microvascular rarefaction in the parafoveal SVP mixed (diabetic and pre-diabetic) cohort.
Mustafar et al. (2023) [14]	Cross-sectional; n = 84 patients stages 3–5 CKD (n = 28 ESRD not undergoing dialysis)	N = 54 in the overall group	Hypertension (n = 78), Ischemic heart disease (n = 20)	Fundus Imaging +OCT	Retinal vessel tortuosity	Retinal vessel tortuosity was negatively correlated with eGFR (r = −0.22, *p* = 0.044) in the overall cohort. No correlation between tortuosity and DM in the overall cohort.	Increased retinal vessel tortuosity in mixed (diabetic and pre-diabetic) cohort with advanced CKD.
Tang et al. (2024) [19]	Cross-sectional; n = 118 CKD 2-5 (n = 48 ESRD), n = 142 eyes (n = 68 eyes from ESRD patients)	No	Not reported	Fundus imaging + OCTA	Vascular density Choroidal vascular index	Retinal vessel density significantly increased with progression towards ESRD (*p* = 0.001). Choroidal vascular index did not differ significantly across CKD stages (*p* = 0.107)	Compensatory vasodilation or artifact in the retinal vessels. The choroid vessels appear less sensitive to CKD progression.
Tang et al. (2024) [19]	Cross-sectional, study lot of n = 118 CKD 2-5 (n = 48 ESRD), OCTA exam for n = 72 patients (n = 142 eyes), eyes from ESRD patients: n = 68	No		Fundus imaging + OCTA	Fundus retinopathy grade Retinal thickness Choroidal thickness	Severity of fundus damage and retinal thinning correlated with CKD stage (Kendall’s tau-b = 0.494, *p* < 0.001). Risk of fundus injury reduced by 94.8% in CKD stage 3 compared to stage 5. Choroidal thickness did not differ significantly across CKD stages.	Fundus pathology reflects renal decline.

Abbreviations: *CKD* chronic kidney disease; *DM* Diabetes Mellitus; *DVP* Deep Vascular Plexus; *DVP-VD* Deep Vascular Plexus-vessel density; *eGFR* estimated glomerular filtration rate; *ESRD* end-stage renal disease; *FAZ* Foveal avascular zone; *FLV* focal loss volume; *GCC* macular ganglion cell complex; *GCCt* macular ganglion cell complex thickness; *GLV* global loss volume; *HD* Hemodialysis; *OCTA* Optical Coherence Tomography Angiography; *PfRT* parafoveal retinal thickness; *RNFL* retinal Nerve Fiber Layer; *SVP* Superficial Vascular Plexus; *SVP-VD* Superficial Vascular Plexus-vessel density.

**Table 3:** Chronic Retinal and Choroidal Microvascular changes in ESRD patients with DM.

Study (Year)	Study Design/Population (n)	Other Systemic Comorbidities	Imaging Modality	Main Retinal Parameter(s)	Key Findings	Clinical Implication
Hwang et al. (2019) [28]	Retrospective observational study; N = 15 diabetic ESRD patients starting HD (26 eyes)	Hypertension (n = 8)	SD-OCT	Central subfield thickness Subfoveal choroidal thickness	Retinal (*p* = 0.006) and choroidal thickness (*p* = 0.024) significantly decreased within 1 month after starting HD. Central retinal decrease correlated with decrease in BUN (r = 0.481, *p* = 0.013). Macular edema incidence decreased from 69% to 26% (*p* = 0.001)	Macular edema and central subfield thickness decrease in ESRD with improvement in uremia and volume overload after initiation of dialysis.
Nakano et al. (2020) [29]	Prospective observational study; N = 16 diabetic ESRD (30 eyes). N = 8 NDR, N = 16 NDPR, N = 6 PDR)	Not reported	SS-OCTA	Subfoveal choroidal thickness LCVLT	Mean SCT and mean LCVLT decreased significantly after HD initiation (*p* < 0.001 for both.) Decrease was more pronounced compared to non-diabetic group (*p* = 0.049 for SCT decrease, *p* = 0.02 for LCVLT decrease).	Substantial changes in choroidal layer after HD initiation in diabetic ESRD compared to non-diabetic ESRD group, possibly reflecting diabetes-induced choroidal vascular disorders.
Takamura (2020) [30]	Retrospective observational study; n = 70 ESRD patients with DR (132 eyes)	Not reported	OCT	Central retinal thickness	Central retinal thickness decreased significantly for 12 months follow-up after initiating HD (*p* < 0.001) despite lack of specific ocular treatment in 93% of cases. More significant reduction in eyes with DME-type subretinal detachment than in those with spongelike swelling and cystoid macular edema.	Starting HD in ESRD patients with DR/DME is linked to 1-year anatomical (CRT) and functional (BCVA) improvements even without additional ocular treatments (largest anatomical response in SRD-type DME).
He et al. (2024) [31]	Prospective observational study; N = 44 ESRD patients with DR (85 eyes)	Not reported	SS-OCTA	Retinal thickness and volume, subfoveal choroidal thickness and volume, superficial capillary plexus, deep capillary plexus, FAZ	Retinal non-perfusion areas and abnormal retinal microvasculature in all eyes. Enlarged FAZ (100%). DME (n = 17, 20%)-all cystoid ERM (n = 7)	Advanced diabetic retinopathy is common with long-term HD, despite apparent macular stability.

Abbreviations: *BCVA* best corrected visual acuity; *BUN* Blood Urea Nitrogen; *CRT* central retinal thickness; *DME* Diabetic Macular Edema; *DR* diabetic retinopathy; *ERM* epiretinal membrane; *ESRD* end-stage renal disease; *FAZ* foveal avascular zone; *HD* hemodialysis; *LCVLT* large choroidal vessel layer thickness; *NDR* diabetic retinopathy; *NDPR* non-proliferative diabetic retinopathy; *PDR* proliferative diabetic retinopathy; *SS-OCTA* Swept-Source Optical Coherence Tomography Angiography; *SD-OCT* spectral-domain Optical Coherence Tomography; *SRD* serous retinal detachment; *SCT* subfoveal choroidal thickness.

## Data Availability

No new data were created or analyzed in this study. Data sharing is not applicable to this article.

## Abbreviations

The following abbreviations are used in this manuscript:

ACS	Acute Coronary Syndrome
AKI	Acute Kidney Injury
BCVA	Best corrected visual acuity
BUN	Blood Urea Nitrogen
CKD	Chronic Kidney Disease
CRF	Chronic Renal Failure
CRT	Central retinal thickness
CT	Choroidal Thickness
CVD	Cardiovascular disease
DM	Diabetes Mellitus
DME	Diabetic Macular Edema
DR	Diabetic retinopathy
DVP	Deep Vascular Plexus
DVP-VD	Deep Vascular Plexus—vessel density
eGFR	Estimated Glomerular Filtration Rate
ESKD/ESRD	End-Stage Kidney Disease/End-Stage Renal Disease
ERM	Epiretinal membrane
FAZ	Foveal avascular zone
FD-OCT	Fourier Domain Optical Coherence Tomography
FLV	Focal loss volume
GCC	Macular ganglion cell complex
GCCt	Macular ganglion cell complex thickness
GCL	Ganglion cell layer
GCIPL	Ganglion cell–inner plexiform layer
GLV	Global loss volume
HD	Hemodialysis
ILM	Internal limiting membrane
IOP	Intraocular Pressure
IPL	Inner plexiform layer
LC	Lamina Cribrosa
LCVLT	Large choroidal vessel layer thickness
MOPP	Mean Ocular Perfusion Pressure
NDPR	Non-Proliferative Diabetic Retinopathy
NDR	Diabetic Retinopathy
OCT	Optical Coherence Tomography
OCTA	Optical Coherence Tomography Angiography
OPL	Outer plexiform layer
PD	Perfusion density
PDR	Proliferative Diabetic Retinopathy
PfRT	Parafoveal retinal thickness
pRNFL	Peripapillary Retinal Nerve Fiber Layer
RPE	Retinal Pigment Epithelium
RNFL	Retinal Nerve Fiber Layer
RNFLt	Retinal Nerve Fiber Layer thickness
RT	Retinal Thickness
SCT	Subfoveal Choroidal Thickness
SD-OCT	Spectral-Domain Optical Coherence Tomography
SRD	Serous Retinal Detachment
SS-OCTA	Swept-Source Optical Coherence Tomography Angiography
SVP	Superficial Vascular Plexus
SVP-VD	Superficial Vascular Plexus—vessel density
